# Practical Notes on Diagnosis and Treatment

**Published:** 1906-06-16

**Authors:** 


### Practical Notes on Diagnosis and Treatment.

**Treatment of Ringworm.**

In two recent cases of ringworm the use of the glass electrode of the high frequency apparatus and an ointment of ichthyol and resorcin was followed by a perfect cure, in each instance, in the course of 14 days.—*Dr. Arthur Roberts.*

**Apical Bronchiectasis.**

Signs of a cavity at the apex of the lung with absence of tubercule bacilli from the sputum ought to suggest the possibility of apical bronchiectasis—a condition which often fail to secure recognition.—*Dr. H. Batty Shaw.*

**Kernig's Sign in Herpes Zoster.**

In 19 cases of herpes zoster Belbeze found Kernig's sign present in two instances. This suggests that herpes zoster depends on disease of the central nervous system, as Kernig's sign is an evidence of meningeal irritation.—*Lancet,* May 26th, 1906.

**Sub-Acute and Chronic Rheumatism.**

Administration of aspirin (5 grains) with quinine (2 grains) thrice daily for several months is useful treatment in chronic rheumatism of the joints. It relieves pain and improves the general and local nutrition.—*Dr. J. W. Martin.*

**Optic Neuritis in Posterior Basic Meningitis.**

Optic neuritis does undoubtedly occur in posterior basic meningitis, but it is very rare. In many cases there are ill-defined appearances of some change in the fundus, but insufficient to warrant a diagnosis of neuritis.—*Dr. O. Hildesheim.*

**Hæmatemesis without Gastric Ulcer.**

Copious hæmatemesis sometimes occurs without ulceration of the gastric mucous membrane. The patients are chiefly young women, and the bleeding is probably due to general oozing from the mucous membrane of the stomach. In the great majority of such cases the patient makes a good recovery.—*Dr. Hale White.*

**Radium in Rodent Ulcer.**

At the Hospital for Diseases of the Skin, Blackfriars, 23 cases of rodent ulcer have, since 1903, been treated by exposure to radium, and in every instance with success. This method of treatment is now being applied to cases of Paget's Disease, to psorospermosis, and also to superficial carcinoma, and the results so far are excellent. It seems to be agreed that, except when the disease extends to the bone, the radium treatment is a specific for rodent ulcer.—*Lancet,* May 19, 1906.

**Manual Treatment of Sciatica.**

M. Berne advocates what he terms the "knee method" in the treatment of sciatica. The patient is placed on the couch in the dorsal position, and the operator stands on the affected side. If this is the right side the operator flexes his right knee and holds this firmly against the patient's right sciatic notch. He then seizes the extremity of the patient's right lower limb and flexes the thigh on the pelvis with the leg extended. This combination of pressure with progressive stretching is said to give excellent results.—*Lancet,* May 19, 1906.

**Fractures in Tabes Dorsalis.**

Fractures may occur in tabetics from falls and even from strenuous muscular effort; it is often difficult to secure union of the bones.—*Sir Wm. R. Gowers.*

**Angio-Neurotic Œdema.**

Dr. Claud A. P. Truman reports a case of this condition cured by 5-grain doses of quinine salicylate. The patient was an adult male, and the parts affected were the eyelids, tongue, lips, fauces, and œsophagus. Some local benefit followed the application of adrenalin dissolved in glycerine.

**The Action of Adrenalin.**

When injected into a vein adrenalin promptly produces vaso-constriction and a rise of blood-pressure with acceleration of the pulse. But introduced into the stomach, adrenalin is destroyed, and thus exerts no constricting action on the blood-vessels. Even when injected subcutaneously it still produces no effect on the systemic blood-vessels.—*Professor W. E. Dixon.*

**Operation in Exophthalmic Goitre.**

Operation in the early stage of Basedow's disease does not expose the life of the patient to any danger. I have not lost one case when the operation could be performed early, even in very bad cases, but I have been very careful to secure judicious medical treatment for these patients for weeks before the operation. A judicious use of very small doses of iodine for a short time, the continuous use of phosphates (sodium phosphate, 2 to 10 grams a day), and absolute mental and bodily rest are the most important features of it.—*Professor Kocher.*

**Arsenic by Subcutaneous Injection.**

In chronic glandular enlargements, and in other cases where arsenic has to be given continuously, and for prolonged periods, the development of gastritis and consequent intolerance often forces suspension of the remedy. To meet this difficulty arsenic can sometimes be given successfully by the hypodermic method. A serviceable preparation for this purpose is the solution of arsenate of iron prepared by Messrs. Squire, of Oxford Street, W. There are two strengths, and the dose of each is 1 c.cm. It is well to commence with the weaker of the two, giving at first a dose on every second day. *Dr. James Galloway.*

**Treatment of Diphtheria.**

Mr. G. L. Thornton advocates moderate or small doses of antitoxin rather than the large initial doses advised by some writers. He also advises sprays to the throat and nasal syringing, as there is often a mixed infection. For these purposes glycerine of boric acid is an excellent application. A spray composed of lime water, benzoate of sodium, and phenol is specially useful in cases with laryngeal symptoms. Internally perchloride of iron with quinine and glycerine should be ordered. Complete rest in bed for the first month, the use of a pillow being forbidden, is a valuable routine measure against the supervention of cardiac failure.—*Lancet,* May 26, 1906.

